# Effect of Spironolactone on Diabetic Nephropathy Compared to the Combination of Spironolactone and Losartan

**DOI:** 10.5812/numonthly.12148

**Published:** 2014-01-14

**Authors:** Atieh Makhlough, Zahra Kashi, Ozra Akha, Ehsan Zaboli, Jamshid Yazdanicharati

**Affiliations:** 1 Diabetes Research Center, Mazandaran University of Medical Sciences, Sari, IR Iran; 2 Molecular and Cellular Biology Research Center, Mazandaran University of Medical Sciences, Sari, IR Iran; 3 Mazandaran University of Medical Sciences, Sari, IR Iran; 4 Department of Biostatistics and Epidemiology, Mazandaran University of Medical Sciences, Sari, IR Iran

**Keywords:** Diabetic Nephropathy, Diabetes Mellitus, Type 2, Albuminuria, Spironolactone

## Abstract

**Background:**

Diabetic nephropathy is the most important cause of end stage renal disease (ESRD). Aldosterone is involved in renal damage through induction of fibrosis, inflammation and necrosis in the kidney tissue. Previous studies have demonstrated that the combination of angiotensin receptor blocker (ARB) and spironolactone (an anti-aldosterone drug) are efficient for albuminuria reduction.

**Objectives:**

This study was designed to evaluate the effect of spironolactone alone on diabetic nephropathy.

**Patients and Methods:**

In this double blind randomized clinical trial, 60 type II diabetic patients with microalbuminuria were enrolled. They were divided into two groups: case group (spironolactone 25 mg and placebo, 30 cases) and control (spironolactone 25 mg plus losartan 25 mg, 30 cases). The treatment success rate (more than 50% reduction in microalbuminuria) was compared between the two groups.

**Results:**

After three months, successful treatment was seen in 70% (95% CI: 52 - 83) and 83.3% (CI 95%: 66 - 93) of case and control groups, respectively (P = 0.4). Mean ± SD of serum potassium levels after three months in case and control groups were 4.56 ± 0.38 and 4.39 ± 0.34 mEq/L, respectively (P = 0.08). Mean ± SD of systolic blood pressures in case and control groups were 129.67 ± 9.4 and 130.97 ± 9.4 mmHg, respectively (P = 0.6). Mean ± SD of serum creatinine levels at the end of the study were 0.95 ± 0.15 in case and 0.90 ± 0.22 mg/dL in control group (P = 0.4).

**Conclusions:**

Spironolactone alone is as effective as the combination of spironolactone and losartan on albuminuria reduction in type 2 diabetic patients and can be used alone as an effective drug for diabetic nephropathy.

## 1. Background

Diabetic nephropathy is the most significant cause of end-stage renal disease (ESRD) and the main cause of mortality and morbidity in diabetic patients. The prevalence of nephropathy in diabetes mellitus (DM) type 1 is more than DM type 2, but due to the greater number of patients presenting DM type 2, its nephropathy rate is as high as type 1 DM (1). Diabetic nephropathy is characterized by albuminuria and usually associated with hypertension, high incidence of cardiovascular morbidity and mortality and progressive renal dysfunction. The main poor prognostic factors include uncontrolled blood pressure and blood sugar, dyslipidemia and high level of proteinuria (2-4). Diabetic nephropathy finally leads to renal failure and necessitates the replacement therapy; thus, scientists are always looking forward to finding the cause of proteinuria and also solutions to slow down its progression (1, 5).

Urinary albumin excretion (albuminuria) is one of the important risk factors for the progression of renal disease to ESRD (1-4, 6). Therefore, control of microalbuminuria can slow down the progression of nephropathy (7-12). Interventional studies have demonstrated that interruption of renin-angiotensin-aldosterone system by angiotensine-converting enzyme inhibitors (ACEI) or angiotensin receptor blockers (ARB) and renin inhibitors can be extremely helpful for decelerating the progression of renal disease (7-9, 13); but after a while, the aldosterone level (the last product of the renin-angiotensin-aldosterone system) increases to its original level due to the aldosterone escape phenomenon. This phenomenon that occurs in about 40% of patients with diabetic nephropathy, usually happens in long-term ACEIs and ARBs consumers (12, 14). Aldosterone acts as a renal injury mediator through inflammation induction, fibrosis and necrosis in the kidney tissue (15-17). It is assumed that aldosterone reduces the BNP7 expression, and down-regulation of BMP7 expression is one of the early events in diabetic nephropathy (18, 19). Therefore, it is proposed that usage of ACEIs and ARBs alone cannot prevent the aldosterone effects (1, 20). Some studies have reported that adjuvant therapy with aldosterone receptor blockers such as spironolactone can be effective for the albuminuria improvement (1, 12, 21-23).

## 2. Objectives

Present study was performed to evaluate the effect of spironolactone alone compared to the combination of spironolactone and losartan on albuminuria reduction in type II diabetic patients.

## 3. Patients and Methods

This study was a double-blinded randomized clinical trial, performed in the DM health care centers of Sari, Iran from 2008 to 2011. The study was registered in IRCT (Iran) with the following registration code: IRCT 138806211241N2. Sixty patients with type 2 diabetes mellitus, suffering from diabetic nephropathy, were enrolled in the study. The inclusion criteria were age range of 25 to 75 years, HbA1c < 9% and random urinary albumin to creatinine ratio of 20 - 200 mg/gr Cr in two random measurements with a month interval. If only one of the two microalbumiuria tests was positive, it would be repeated the next month. Exclusion criteria were: diastolic and systolic blood pressures more than 100 and 160 mmHg, respectively; serum potassium level > 5.5 meq/L; prior acute myocardial infarction (MI) or stroke during the preceding six-month period; taking proteinuria-affecting medications (corticosteroids, NSAIDs, immunosuppressant drug); renovascular disease; collagen vascular disease; obstructive uropathy; alcohol and substance abuse; pregnancy or lactation.

This study had two phases; screening and treatment. During the screening phase, patients were selected according to the inclusion criteria and then eligible patients were entered into the treatment phase. Five milliliters of fasting blood was taken for the serum creatinine, potassium, and glycosylated hemoglobin (HbA1C) assessments, and the tests were repeated 4 and 12 weeks after the intervention. Limitation of protein consumption (0.6-0.8 g/kg/d) was advised during the study. The samples were assigned to case or control group through the RANDBETWEEN function of Microsoft Excel software. Case group comprised 30 diabetic patients who took spironolactone 25 mg once daily plus half a tablet placebo twice a day. Control group took spironolactone 25 mg once daily plus losartan 12.5 mg twice a day. The intervention phase lasted for 12 weeks.

Patients’ drugs consumption quality was evaluated by counting the remaining tablets at the end of each month. Albuminuria was measured in the beginning, after 4 weeks and at the end of the intervention. For these measurements, immunotorbidometric assay was performed by prestige 24i automated clinical chemistry analyzer (auto analyzer, Japan), using the Pars Azmon kit (Iran). Patients were visited every 4 weeks and their serum potassium levels were measured. Serum potassium level was measured by Alfa Wassermann Starlyte III electrolyte analyzer and serum creatinine was assessed by Cobas Integra 400 (Roche Diagnostics GmbH, Germany), using the Creatinine Plus kit (Roche Diagnostics GmbH, Germany).

Spironolactone dosage was reduced by half if the serum potassium level increased more than 5.5 mmol/L or systolic BP decreased less than 90 mmHg. The study was approved by the Mazandaran University of Medical Sciences Ethics Committee (Ethic code: 88-89) and informed consent was obtained from all the participants.

### 3.1. Statistical Analysis

The data were described as mean ± SD (standard deviation). Independent sample t-test was used to compare the baseline and after intervention levels of microalbuminuria between case and control groups. Repeated measures analysis was applied for evaluation of the treatment effect as well as the trend of treatment in each group. The treatment success rate was reported as 95% confidence level. P value < 0.05 was considered statistically significant.

## 4. Results

Twenty-three (76.7%) of the case and 22 (73.3%) of the control group were female (P = 0.8). One patient of the case group was dropped out from the study because of not using the medications regularly. Mean age of the patients was 51.78 ± 11.39 years. Fifty patients (83.3%) were under oral antidiabetic agents and 10 (16.7%) were taking insulin. Patients under treatment with insulin were 13.3% of the case and 20% of the control group (P = 0.5). Table 1 shows the baseline information in two groups.

**Table 1.** Descriptive Data Before Intervention in Two Groups of Spironolacton + Losartan and Spironolacton + Placebo

Variable	Spironolacton + Losartan, Mean ± SD	Spironolacton + Placebo, Mean ± SD	P Value
**Age, y**	51.2 ± 12.29	52.3 ± 10.61	0.8
**DM^a^ duration, y**	8.66 ± 5.39	6.33 ± 5.19	0.1
**GFR^a^, mL/min**	115.6 ± 23.5	112.5 ± 25.6	0.6
**FBS^a^, mg/dL**	152.63 ± 44.43	147.4 ± 44.66	0.6
**HbA1c, No. (%)**	6.93 ± 0.86	7.2 ± 0.79	0.2
**Systolic BP, mmHg**	136.03 ± 4.90	132.33 ± 11.18	0.1
**Diastolic BP, mmHg**	82.40 ± 7.89	80.43 ± 6.96	0.3
**Urine albumin, mg/g**	102.03 ± 51.98	81.11 ± 51.9	0.1
**Potassium, meq/L**	4.32 ± 0.35	4.49 ± 0.31	0.05

^a^ Abbreviations: DM, diabetes mellitus; FBS, fasting blood sugar; GFR, glomerular filtration rate.

The treatment success rate (more than 50% reduction in microalbuminuria) 1 month after the intervention was 66.7% and 56.7% in the case and control groups, respectively (P = 0.4). After 3 months, this rate was 70% in the case and 83.3% in control group (P = 0.4) (Table 2). The treatment success rate was not different between two sexes. After 4 weeks of intervention, microalbuminuria disappeared in 50% of cases and 33.3% of controls (P = 0.2). Sixty percent of the control and 46.7% of the case group did not have microalbuminuria at the end of the study (P = 0.2).

**Table 2.** The Treatment Success Rate in Two Groups of Spironolacton + Losartan and Spironolacton + Placebo

	Spironolcton + Losartan	Spironolacton + Placebo	P Value
**Mal**e			
After 4 weeks	75% (CI 95%: 41-93)	71.4% (CI 95%: 36-92)	0.9
After 12 weeks	100% (95%CI: 48-100)	85.7% (95%CI: 49-97)	0.3
**Female**			
After 4 weeks	50% (CI 95%: 31-69)	65.2% (CI 95%: 45-81)	0.3
After 12 weeks	77.3% (95%CI: 57-90)	65.2% (95%CI: 45-81)	0.5
**Total**			
After 4 weeks	56.7% (CI95%: 39-73)	66.7% (CI 95%: 49-81)	0.4
After 12 weeks	83.3% (95%CI: 66-93)	70% (95%CI: 52-83)	0.4

**Figure 1.**
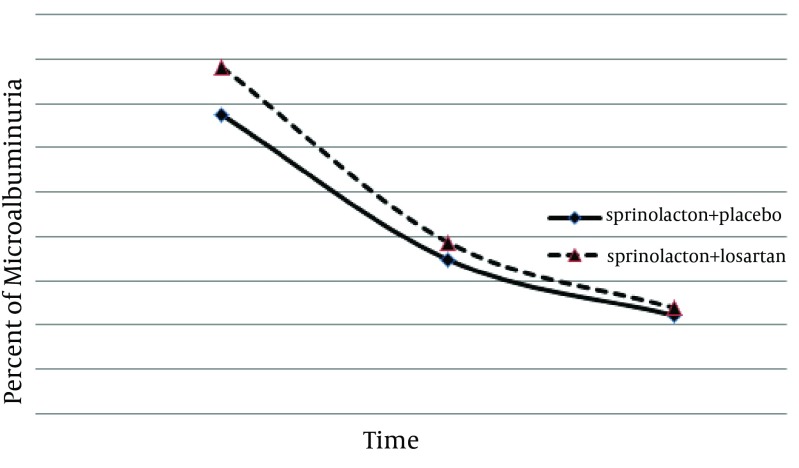
The Trend of Treatment Response in Two Groups of Spironolacton + Losartan and Spironolacton + Placebo

There were no significant increases in the serum creatinine or potassium levels of any patient. Microalbuminuria, serum creatinine level, and systolic/diastolic blood pressures were not different between the two groups after the study (Table 3). Using the repeated measures analysis, a significant therapeutic effect was observed in both groups (F = 7.45, P = 0.009) but there was no significant difference between the two treatment methods regarding albuminuria reduction (F = 0.12, P = 0.7) (Figure 1). 

**Table 3.** Comparison of Microalbuminuria, Serum Creatinine Level, Serum Potassium Level, and Systolic/Diastolic Blood Pressures Between Case and Control Groups Quantitative Data After the Treatment

Variable	Spironolacton + Losartan, Mean ± SD	Spironolacton + Placebo, Mean ± SD	P Value
**Urine albumin, mg/g**			
After 4 weeks	48.25 ± 50.05	46.3 ± 45.6	0.87
After 3 months	33.98 ± 35.3	32.24 ± 38.7	0.86
**Urine albumin change, mg/g**			
After 4 weeks	-53.9 ± 36.0	-42.8 ± 48.7	0.3
After 3 months	-60.4 ± 63.4	-60.4 ± 30.5	1.00
**GFR^a^, mL/min**			
After 4 weeks	116.5 ± 26.2	108.0 ± 17.9	0.2
After 3 months	115.4 ± 24.1	107.5 ± 17.2	0.2
**GFR change, mL/min**			
After 4 weeks	0.9 ± 15.5	-4.6 ± 15.0	0.2
After 3 months	-0.3 ± 21.0	-4.5 ± 18.6	0.4
**Systolic BP after 3 months, mean ± SD, mmHg**	130.97 ± 9.4	129.67 ± 9.4	0.6
**Diastolic BP after 3 months, mmHg**	77.97 ± 8.4	77.59 ± 6.63	0.9
**Potassium after 3 months, meq/L**	4.39 ± 0.34	4.56 ± 0.38	0.08

^a^ Abbreviation: GFR, glomerular filtration rate.

## 5. Discussion

In this study, we found that in diabetic patients, treatment with spironolactone alone has the same effect as combination therapy with spironolactone and losartan on microalbuminuria reduction. ACEIs and ARBs effectively reduce proteinuria and postpone renal disease progression in diabetic and non-diabetic patients (21, 24). Anti-proteinuria effects of ACEIs and ARBs decrease in long-term follow-ups due to the aldosterone escape phenomenon, emerged in about half of diabetic patients (14). Clinical and experimental evidences show that aldosterone can cause nephrosclerosis progression and renal fibrosis in patients with diabetes and hypertension (2, 9, 12, 14). ACEIs and ARBs failure in long term suppression of aldosterone is the main cause of their defeat in proteinuria management (1, 14, 25). So the blockage of mineralocorticoid receptors with spironolactone can prevent kidney and heart damages (26). The exact mechanism of antiproteinuria effect of spironolactone is not clearly recognized. Although spironolactone is a diuretic drug, the blood pressure of patients in this study did not significantly change after the treatment, so it seems that its blocking effect on the mineralocorticoid receptors is distinct from its hemodynamic impact. In the present case, antiproteinuria effect of spironolactone was seen after 4 weeks of treatment without changes in the blood pressure. Similar findings were also reported in other studies. Effect of spironolactone in overt diabetic nephropathy was evaluated in a randomized, double blind crossover study. Rossing et al. enrolled 21 patients under the maximum dose of ACEI or ARB. Albuminuria was measured after 8 weeks. It was revealed that adding low-dose spironolactone has additional reno-cardiovascular protectional influences without significant side effects (22). In our study, monotherapy with spironolactone showed similar clinical efficiency as combination therapy with spironolactone and losartan. Rossing et al. evaluated overt diabetic nephropathy, but we studied microalbuminuria; thus the difference may affect the response rate. Rachmani et al. compared the efficacy of spironolactone alone as well as in combination with ACEI (cilazapril). In the mentioned study which only assessed diabetic females, spironolactone alone was effective in reducing albuminuria, which was similar to our results; however, the combination of spironolactone and cilazapril was more effective than spironolactone alone in their report (21). Davidson et al. studied the effect of spironolactone and ACEI on albuminuria in 11 micro and 13 macroalbuminuria subjects. They suggested that addition of spironolactone to ACEI can result in a greater microalbuminuria reduction (27).

Type IV collagen is a component of glomerular basement membrane and mesangial matrix, production of which can be induced by aldosterone. Its urinary appearance can be a reflection of its production level (28) and it causes progressive renal fibrosis. ACEIs alone do not have remarkable inhibitory effects in this process (29), while spironolactone reduces the progression of renal fibrosis (18). Furthermore, it has been declared that in spite of the maximum antiproteinuria effect of ACEI, urinary excretion of TGF-β1 (transforming growth factor) is high in these patients. TGF-β1 is a profibrotic cytokine that stimulates the protein synthesis in the extracellular matrix (30). A study on the diabetic mice has also shown that treatment with ACEI could not prevent its high production in glomeruli (31). In comparison, spironolactone could reduce the TGF-β1 secretion in cyclosporine-induced nephrotoxicity (32, 33). Therefore, it seems that mineralocorticoid receptor blockers such as spironolactone are preferred to ACEIs, for TGF-β1 inhibiting activity.

Hyperkalemia is one of the complications of spironolactone. It is dependent on drug dosage; so low dose spironolactone can provide a safe margin (22). In our study, the potassium level was carefully monitored, and only one patient (in the spironolactone-losartan group) had a potassium level of over 5.5 mEq/L at the end of the study.

Our study had some limitations. Its population was pretty small and the level of microalbuminuria was different between the two groups before the intervention, but it was not statistically significant. Also, we just evaluated microalbuminuria in diabetic patients. According to our findings, we suggest that spironolactone alone can be effective in treatment of patients with diabetic nephropathy. More studies need to be done to establish the long-term beneficial clinical effects of spironolactone alone in different stages of diabetic nephropathy.
